# Source Localization with Acoustic Sensor Arrays Using Generative Model Based Fitting with Sparse Constraints

**DOI:** 10.3390/sl21013781

**Published:** 2012-10-15

**Authors:** Jose Velasco, Daniel Pizarro, Javier Macias-Guarasa

**Affiliations:** Department of Electronics, University of Alcalá, Campus Universitario s/n, 28805, Alcalá de Henares, Madrid, Spain; E-Mails: pizarro@depeca.uah.es (D.P.); macias@depeca.uah.es (J.M.-G.)

**Keywords:** acoustic localization, microphone array sensors, sparse modeling, optimization techniques

## Abstract

This paper presents a novel approach for indoor acoustic source localization using sensor arrays. The proposed solution starts by defining a generative model, designed to explain the acoustic power maps obtained by Steered Response Power (*SRP*) strategies. An optimization approach is then proposed to fit the model to real input *SRP* data and estimate the position of the acoustic source. Adequately fitting the model to real *SRP* data, where noise and other unmodelled effects distort the ideal signal, is the core contribution of the paper. Two basic strategies in the optimization are proposed. First, sparse constraints in the parameters of the model are included, enforcing the number of simultaneous active sources to be limited. Second, subspace analysis is used to filter out portions of the input signal that cannot be explained by the model. Experimental results on a realistic speech database show statistically significant localization error reductions of up to 30% when compared with the *SRP-PHAT* strategies.

## 1. Introduction

The development and scientific research in perceptual systems has notably grown during the last decades. The aim of perceptual systems is to automatically analyze complex and rich information taken from different sensors. These systems stem from basic sensor technologies, reaching the knowledge frontier in signal processing and pattern recognition research areas.

On top of perceptual systems, the idea of using sensors to analyze the real world has emerged in different scientific disciplines such as “ubiquitous computing” [1], “smart rooms” [2] or “intelligent spaces” [3]. All these disciplines lay stress on the idea of systems with interaction capabilities that can analyze human activities and provide services.

A basic but important milestone inside these disciplines is the development of sensor technologies able to localize humans in indoor environments. Localization of humans has a tremendous potential impact in diverse applied fields, opening new ways in how humans interact with machines. One important factor in indoor localization is the user awareness of the sensors used. Non-invasive technologies are preferred in this context, so that no electronic or passive devices are to be carried by humans for localization. The two non-invasive technologies that have been mainly used in indoor localization are those based on video systems and acoustic sensors.

Video systems provide very rich information at a low cost on the sensor side. However, video analysis is a complex problem and needs a lot of effort to build robust and reliable systems. In recent years, there are many publications focused on video-based indoor localization systems for humans [4,5], robots [6], and object recognition systems [7].

Acoustic sensors give also very rich information as humans communicate mainly with speech. As in video, there is also a considerable amount of publications focused on obtaining the exact position of any active acoustic source in a scene [8,9]. Video and audio technologies are in fact very complementary in many ways [10].

This paper focuses on audio-based localization in a very general scenario, where unknown wide-band audio sources (e.g., human voice) are captured by a set of microphone arrays placed in known positions. The main objective of the paper is to use the signals captured by the microphone arrays to automatically obtain the position of the acoustic sources detected. Especially relevant in practice are the methods based on computing the Steered Response Power (*SRP*) [11] of the signals captured in microphones arrays. These approaches have proved to be successful for localization in reverberant and noisy scenarios [12].

This paper proposes a simple generative model to explain *SRP* measurements in environments equipped with any combination of microphone arrays. The main contribution of the paper is to use an optimization approach to fit the generative model to noisy *SRP* data, exploiting the fact that only a few speakers are expected to be active at the same time. This simple idea is modeled with sparse constraints in the optimization cost, and combined with subspace filtering. The paper shows that this model-based approach can be used to notably improve the localization results of the state-of-the-art methods based on *SRP-PHAT*. Although this proposal is developed and evaluated for speech signals, the authors believe that it is general enough to be easily extended to other wideband and narrowband acoustic signals.

### 1.1. Paper Structure

The paper is structured as follows. In Section 2 we provide an extensive study of the state-of-the-art in acoustic source localization and optimization methods. Section 3 describes the proposed generative model and Section 4 deals with the optimization strategy to fit the model to real data. The experimental evaluation is detailed in Section 5, and Section 6 summarizes the main conclusions and contributions of the paper and gives some ideas for future work.

## 2. State of the Art

### 2.1. Acoustic Source Localization

The acoustic source localization methods are the starting point of other techniques like speech enhancement using beamforming. Therefore, acoustic source localization has received significant attention lately as a mode of automatic tracking of persons and as a complement to other existing alternatives of tracking, e.g., the CHIL (Computer in Human Interaction Loop) project [10].

Many approaches exist in literature and all of them use microphone arrays as a non-intrusive method. These can roughly be divided in three categories [8,9]: time delay based, beamforming based, and high-resolution spectral-estimation based methods.

The first methods are based on estimating the time delay of signals relative to pairs of spatially separated microphones. Assuming uncorrelated, stationary Gaussian signal and noise with known statistics and not multi-path, the maximum likelihood (ML) time-delay estimate is derived from a SNR-weighted version of the Generalized Cross Correlation (GCC) function [13]. In a second step, the time-difference of arrival information is combined with knowledge of the microphones' positions to generate a ML spatial estimator made from hyperbolas intersected in some optimal sense [8,9].

An accurate estimation of the time delay is essential for a good performance of this *time delay of arrival* (TDOA) methods. Since coherent noise and multi-path due to reverberation are the two major sources of error in time delay estimation, different approaches have been proposed to deal with them. A basic method consists in making the GCC function more robust, de-emphasizing the frequency-dependent weighting. The Phase Transform (PHAT) [13] is one example of this procedure that has received considerable attention as the basis of speech source localization systems due to its robustness in real world scenarios [14].

Beamforming based techniques [15] attempt to estimate the position of the source, maximizing or minimizing a spatial statistic associated with each position. For instance, in the Steered Response Power (*SRP*) approach, which is the simplest beamforming method, the statistic is based on the signal power received when the microphone array is steered in the direction of a specific location. Therefore, the position of the source is supposed to be consistent with the position corresponding to the maximum estimated signal power

*SRP-PHAT* is a widely used algorithm for speaker localization based on beamforming. It was first proposed in [11] and is a beamforming based method that combines the robustness of the steered beamforming methods with the insensitivity to signal conditions afforded by the Phase Transform (PHAT). The classical delay-and-sum beamformer used in *SRP* is replaced in *SRP-PHAT* by a filter-and-sum beamformer using PHAT filtering to weight the incoming signals. In this paper, the term *SRP* will be used interchangeably with *SRP-PHAT*.

The advantage of using PHAT is that no assumptions are made about the signal or room conditions [16], and this is the reason for the robustness of the *SRP-PHAT* method in reverberant scenarios, where the source is unknown. *SRP-PHAT* is usually defined as a reference standard for source localization, because of its simplicity and robustness in reverberant and noisy environments, being a widely used algorithm for speaker localization [17–21].

The Minimum Variance Distortionless Response (MVDR), also called Capon's method, is another beamforming based approach which takes advantage of the estimated signal and noise parameters. These parameters are used to carry out optimal beamforming techniques in order to minimize the measured power from noise and sources located in other positions. However, MVDR has a poor performance in the presence of reverberation, because it introduces a new trade-off between de-reverberation and noise reduction [22].

In [23,24], a unified maximum likelihood framework is presented, which is equivalent to forming multiple MVDR beamformers along multiple hypothesis directions and picking the output direction which results in the highest SNR [24]. Apparently, it outperforms *SRP-PHAT* in reverberant real scenarios.

The spectral estimation based methods, like the popular multiple signal classification algorithm (MUSIC) [25], exploit the spectral decomposition of the covariance matrix of the incoming signals for improving the spatial resolution of the algorithm in a multiple sources context. These methods tend to be less robust than beamforming methods [9], and are very sensitive to small modeling errors.

Unlike *SRP* and its derivatives, incoherent signals are assumed by MUSIC, but in real scenarios with speech sources and reverberation effects, the incoherence condition is not fulfilled, making the subspace-based techniques problematic in practice.

The work presented in this paper uses *SRP-PHAT* as the base to develop a generative model to explain real data, and the experimental results are compared against *SRP-PHAT*.

### 2.2. Sparse Representation of Signals

Many areas of science share the principle of parsimony as the central criterion: the simplest explanation of a given phenomenon is preferred over more complicated ones. This brilliant idea has been recently applied to the representation of signals using overcomplete basis sets, sometimes called dictionaries in the machine learning discipline. As a difference with respect to traditional basis functions (e.g., Fourier basis functions), overcomplete dictionaries have more degrees of freedom than those necessary to represent the signal. The mathematical tool to impose parsimony in the representation of a signal, when several choices are available, is given by imposing the so-called sparse constraints. The basic idea is to use the least amount of coefficients to represent a signal with the basis functions. Sparse constraints, if they are applicable, allow to beat up several theoretical barriers in signal compression and representation [26,27].

The sparsity is imposed mainly by using optimization approaches, where the *l_0_* norm (defined as the number of non-zero elements in the vector) is the usual way to impose sparsity to vectors [27].

Most of the problems in which sparsity is included using the *l*_0_ norm are very difficult to solve. Several methods have been proposed to find sparse representations, including brute force approaches as well as more computationally efficient approximate methods such as “nonlinear programming” [28], and greedy pursuit [29–31]. Among all approximate solutions, *l*_1_ norm based convex relaxations have flourished in the literature. The Basis Pursuit method [32,33], originally introduced by [34] almost 40 years ago but revisited with a profound theoretical study in the past decade, can be highlighted due to its intensive use in the modern compressive sensing techniques [26,27]. These methods provide very effective polynomial time algorithms that, under certain circumstances, are even equivalent to the original *l*_0_ based problems [27,33].

### 2.3. Sparse Source Localization

In the last few years, sparse techniques explained above have been applied to the source localization problem in very different fashions.

In [35] a localization approach based on sensor arrays is proposed. The signal obtained in each sensor is expressed as a linear combination of an attenuated and phase shifted version of the original and known signals emitted by the source. This conditions form an overcomplete linear model, where the position of the sources is given thanks to the sparse constraints. Also in [35] they propose to use *singular value decomposition* (SVD) to reduce problem size and filter noise in problems using multiple time samples.

The work presented in this paper includes sparse and SVD decompositions for acoustic source localization but the objectives (unknown source signals) and the way these techniques are applied are very different to those of [35]. Our proposal works in the *SRP-PHAT* acoustic power maps, while [35] operates at the sensor signal level.

Numerous modifications of the ideas proposed in [35] has been further developed. For example, in [36] an adaptive algorithm to dynamically adjust both the overcomplete basis and the sparse solution is proposed. Also, the concept of Compressive Sensing [27] has been used in order to perform a distributed localization reducing the information transmitted between sensors. Nevertheless, the sparse source localization algorithms discussed above do not perform well and are not properly tested in real acoustic reverberant environments due to input signals coherence caused by multipath.

In acoustic environments, sparse *l*_1_ relaxations are employed to model the room acoustically using only a reduced number of microphones in [37]. However, only simple rooms (four walls and ceiling) can be modeled, and a loudspeaker emitting a known sound pattern is required. Using this technique in a previous training step has been proved to be useful to improve source localization [38].

Recently, a novel technique for source localization in reverberant environments using wavefield sparse decomposition has been proposed in [39]. However, although it shows promising performance, the experimental results are only based on simulations and narrowband signals, which makes their approach not applicable to speech signals, which is our target scenario.

## 3. Model Proposal

### 3.1. Notation

Real scalar values are represented by lowercase letters (e.g., *δ*). Upper-case letters are reserved to define vector and set sizes (e.g., vector **x** = (*x*_1_, ċ, *x_N_*)^┬^ is of size *N*). Vectors are by default arranged column-wise and are represented by lowercase bold letters (e.g., **x**). Matrices are represented by uppercase bold letters (e.g., M). The *l_p_* norm (*p* > 0) of a vector is depicted as ‖.‖*_p_*, e.g., ${\left\|x\right\|}_{p}={\left({\left|x_{1}\right|}^{p}+\cdots +{\left|x_{N}\right|}^{p}\right)}^{\frac{1}{p}}$, where |.| is reserved to represent absolute values of scalars. Special cases are the *l*_0_ norm, written ‖.‖_0_ and defined as the number of non-zero elements in the vector, and the *l*_∞_ norm, written ‖.‖_∞_ and defined as the maximum value of the vector components. The *l*_2_ norm ‖.‖_2_ will be written by default as ‖.‖ for simplicity. Calligraphic fonts are reserved to represent sets (e.g., ℝ for real or generic sets *G*).

### 3.2. Interpretation of the *SRP-PHAT* Estimations

Assume we have equipped a certain indoor environment with a set of *N* different microphone pairs distributed in some fashion in three-dimensional known positions. All pairs of microphones are described as elements in a set P = {**p**_1_, **p**_2_, …, **p**_n_}, where **p***_j_*, = (**m***_j_*, **m**′*_j_*) is composed of two three-dimensional vectors, **m***_j_*, and **m**′*_j_*, describing the spatial location of the microphones in pair *j*.

The three-dimensional space where acoustic sources are to be localized is discretized using a finite set of Q spatial locations Q = {**q**_1_, **q**_2_, …, **q***_Q_*}, where **q***_k_* is a three-dimensional vector **q***_k_* = (*q_kx_*, *q_ky_*, *q_kz_*)^┬^

The classical *SRP-PHAT* method constructs a statistic *srp*(**q***_k_*), **q***_k_* ∈ Q based on the steered power received by all pairs of microphones from each spatial location. Simplifying the mathematical description of the *SRP-PHAT* formulation of [11] and applying the summation over all microphone pairs, we can write

(1) $$\text{srp}(q_{k})=2\pi \sum_{\forall p_{j}\in P}c_{j}(\Delta \tau (p_{j},q_{k}))$$

where *c_j_*(Δ*τ*(**p***_j_*, **q***_k_*)) is the generalized cross-correlation (generally applying a PHAT weighting) of the signals acquired by each microphone in the pair **p***_j_*, and

(2) $$\Delta \tau (p_{j},q_{k})=\frac{1}{c}(\|m_{j}-q_{k}\|-\|{m^{\prime }}_{j}-q_{k}\|)$$

is the difference in arrival times of the audio signal to reach microphones **m***_j_*, and **m**′*_j_*, that is, the required delay to steer the microphone pair **p***_j_*, to the location **q***_k_*. In Equation (2)*c* is the sound velocity in air. Note than in the *SRP-PHAT* formulation we do not make any assumption regarding near-field/far-field conditions.

So, Equation (1) shows how the *SRP-PHAT* power estimation for every location *srp*(**q***_k_*) can be calculated as the sum of the cross-correlation functions for all microphone pairs, evaluated at the adequate steering delays (full implementation details of *SRP-PHAT* can be found in [11]). It is thus expected to see high values of *srp*(**q***_k_*) in regions in which active acoustic sources exist.

To provide an easier geometric interpretation, we now restrict the result of the *srp*(**q***_k_*) estimations when only one omnidirectional acoustic source is active at position s = (*s_x_*, *s_y_*, *s_z_*)^┬^, and only one microphone pair, e.g., pair **p***_j_*, is located in the environment. The *SRP-PHAT* power estimation at s can be calculated as:

(3) $$\text{srp}(s)=2\pi c_{j}(\Delta \tau (p_{j},s))$$

From Equation (3), if we define **q***_h_* as the locations in Q for which Δ*τ*(**p***_j_*, **q***_h_*) = Δ*τ*(**p***_j_*, **s**), the corresponding cross-correlation values *c_j_*(Δ*τ*(**p***_j_*, **q***_h_*)) will be identical to *c_j_*(Δ*τ*(**p***_j_*, **s**)), consequently:

(4) $$\begin{array}{ccc} \text{srp}(q_{h})=\text{srp}(s) & \text{if} & \Delta \tau (p_{j},q_{h})=\Delta \tau (p_{j},s) \end{array}$$

**Figure 1.**
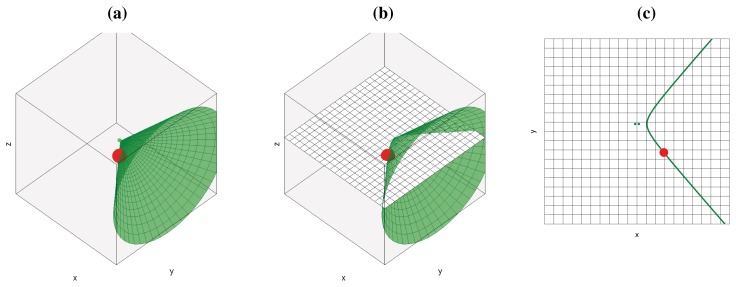
Geometric places with equal *srp*(**q***_h_*) generated for a microphone pair and a single acoustic source (a) 3D hyperboloid; (b) 3D hyperboloid cut by a plane; (c) Resulting 2D hyperbola (cutting hyperboloid by a plane).

For a microphone pair, it can be easily demonstrated that the geometric place of points **q***_h_*, for which the difference in time delays of arrival to the position of two microphones (Δ*τ*(**p***_j_*, **q***_h_*) in our case) is equal to a given fixed value (Δ*τ*(**p***_j_*, **s**) in our case), is one of the sheets of a two-sheeted hyperboloid of revolution, whose foci are located at the microphone locations, as shown in Figure 1(a). If we define ℋ as all the points **q***_h_* in Q that *belong* to the hyperboloid that *passes* through the acoustic source location **s**, the *ideal SRP-PHAT* power estimation for all points in Q will be:

(5) $$\text{srp}(q_{k})=\{\begin{array}{cc} \text{srp}(s) & \forall q_{k}\in ℋ\\ 0 & \text{otherwise} \end{array}$$

Equation (5) is correct if we assume that the environment is not reverberant and the array directivity pattern is perfect (*i.e.*, maximum gain in the steered direction and perfect cancellation in all other directions). We will address the effect of these simplifications in Section 3.3.

Further simplifying, if we restrict the **q***_k_* positions to be located in a plane at a given height in the environment (*q_kz_* = *z*_0_ ∀**q***_k_* ∈ Q), then *srp*(**q***_k_*) can be easily represented as an image that can be interpreted as the scene *acoustic power map*. In this situation, the place of points **q***_k_* with power equal to *srp*(**s**) will be the result of *intersecting* the proper sheet of the hyperboloid of revolution with a plane parallel to the environment floor at *z*_0_, and the generated geometric figure obtained will be a hyperbola.

As an example, if we consider the case of microphone pair **p***_j_*, composed of microphones **m***_j_* = (−*f*, 0, 0) and **m**′*_j_* = (*f*, 0, 0), and given a time difference of arrival $\Delta \tau (p_{j},s)=\frac{1}{c}(\|m_{j}-s\|-\|{m^{\prime }}_{j}-s\|)$ for a speaker position s, the feasible acoustic source locations **q***_h_* = (*x*, *y*, *z*) ∈ Q are those which satisfy the following expression (from Equations (2)–(4)):

(6) $$\Delta \tau (p_{j},q_{h})=\frac{1}{c}(\|m_{j}-q_{h}\|-\|{m^{\prime }}_{j}-q_{h}\|)=\Delta \tau (p_{j},s)$$

Condition (6) defines the place of feasible locations **q_h_** to be located in one sheet of the following two-sheeted hyperboloid of revolution (shown in Figure 1(a)):

(7) $$\frac{x^{2}}{a^{2}}-\frac{y^{2}}{b^{2}}-\frac{z^{2}}{b^{2}}=1$$

where *a* and *b* are related to the corresponding time difference of arrival Δ*τ*(**p***_j_*, **s**) and the microphones position through the following expressions:

(8a) $$a=c\Delta \tau (p_{j},s)/2$$

(8b) $$b^{2}=f^{2}-a^{2}$$

Figure 1(c) shows the hyperbola that results from intersecting the hyperboloid with a plane, as shown in Figure 1(b).

**Figure 2.**
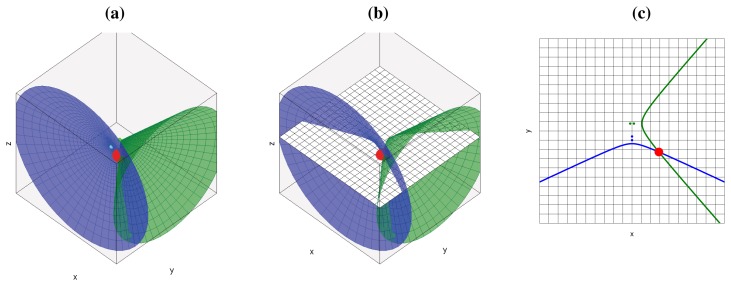
Geometric places generated for two microphone pairs and a single acoustic source (a) 3D hyperboloids; (b) 3D hyperboloids cut by a plane; (c) Resulting 2D hyperbolas (cutting hyperboloids by a plane).

If we add additional microphone pairs, each of them will generate a new hyperboloid/hyperbola, all passing through the geometric location of the active acoustic source, as shown in Figure 2(a) for the 3D case and Figure 2(c) for the 2D case (cutting the hyperboloids by a plane as shown in Figure 2(b)). Using additional microphone pairs will allow us to disambiguate the actual position of the acoustic source, searching in the intersection of all hyperboloids/hyperbolas.

The final conclusion of this section is that, given some simplifications, for every active acoustic source and every microphone pair, we will see hyperbolic regions of *constant* acoustic power values in the acoustic power map generated by the ideal *SRP-PHAT* estimations. All the contributions for every acoustic source and every microphone pair will sum up to build the complete acoustic power map for the given situation.

### 3.3. Considerations in Real-World Scenarios

**Figure 3.**
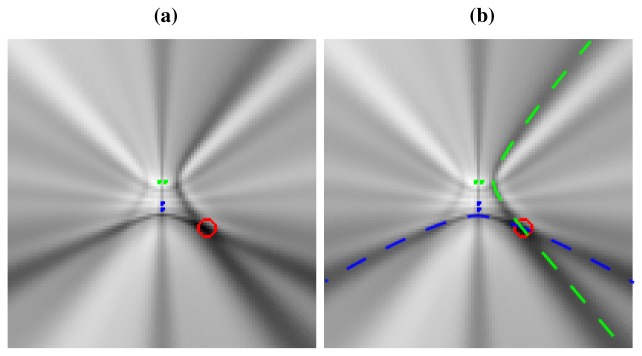
Real *SRP-PHAT* power map generated for a single speaker located in the red circle with two microphone pairs (blue and green dots), (a) Plain power map; (b) Superimposing ideal hyperbolas that should be generated by the single speaker.

The simplifications established in this discussion (namely, only one omnidirectional acoustic active source, an ideal directivity pattern for the acoustic sensor array, and a non-reverberant environment) are far from being admissible in a real world scenario and deserve an additional comment:

- Non-omnidirectionality of the acoustic active source: Previous studies such as [40] and [41] show that human speakers do not radiate speech uniformly in all directions. The impact of this assumption in our *SRP-PHAT* interpretation would lead to hyperbolic regions with different power estimations, but this effect is also present in the current formulation, as the distance between the acoustic source and the microphone varies with the source position. The use of the PHAT transform that *whitens* the correlation of the input signals alleviates this problem, as the module is not taken into account.
- Reverberant environments: If the localization system operates in a reverberant environment, new hyperbolic regions, not initially predicted by just the position of the acoustic source, will appear. Room acoustic simulation techniques could help in improving the ability to also take into account these regions [42,43]. These *false* active regions actually complicate the accurate location estimation, but the problem is alleviated as more microphone pairs are taken into account: locations that are not *consistent* for all microphone pairs will tend to attenuate. As we will see in Section 5, our proposal is actually efficient in *denoising* the original *SRP-PHAT* power map, thus leading to better results.
- Non-ideal directivity patterns: The microphone array geometry has a profound impact in the estimation of the cross-correlation functions, as the steered response will perceive energy coming from locations different from the actual acoustic source [44]. This implies that the acoustic power map will not be composed of plain hyperboloids/hyperbolas, but of hyperbolic *regions* spreading from the ideal hyperbolic trajectories, as will be shown in Figure 3(a), described in the next section. There are additional considerations that contribute to this *spreading* effect, related to the fact that the spatial uncertainty in the correlation evaluation increases as we move further from the microphone pairs. This will be addressed also in the next section.

To give a real world example, Figure 3(a) shows a real *SRP-PHAT* image generated by two microphone pairs (blue and green dots in the center of the image) and a single active speaker located at the red circle (the higher the power, the darker the color in the map). Analyzing this image, we can clearly see two high energy, intersecting hyperbolic areas passing trough the speaker location, each one corresponding to each microphone pair. Obviously, the speaker's position corresponds to the place where those hyperbolic areas intersect, as the maximum of the power map is found at this intersection. In general, the higher the number of microphone pairs used, the better the localization performance, as more hyperbolic regions contribute to the power map estimation. In Figure 3(b) the ideal hyperbolas corresponding to each of the microphone pairs have been superimposed to the *SRP-PHAT* map. The power map has been calculated at a plane located 61 cm above the microphone locations, which is why the hyperbolas do *not pass* between the hyperbola's foci—the microphone locations.

This example shows us that in real acoustic power maps, the ideal hyperbolic functions are spread out and blurred, leading to these hyperbolic *areas*, and that additional hyperbolic areas appear, not explainable by just the position of the active acoustic source.

Summarizing, all these non-idealities will generate additional artifacts, additional hyperbolic regions and variations on the standard behavior of these regions in the acoustic power map that are not predicted by the ideal formulation. These non-idealities should be taken into account if we want our model to be as precise as possible. Our thesis is that our proposal, even when no developing a fully realistic model, is powerful enough to extract relevant information given realistic data, as will be shown in Section 5.

### 3.4. Proposal of a *SRP-PHAT* Based Generative Model

Taking into account the previous discussion and results, this section proposes a generative model that is able to explain the acoustic power map generated by *SRP-PHAT* as a sum of basis functions.

Let us define the set of scalar functions ℱ = {*f*(**s***_i_*, **p***_j_*, **q***_k_*)}, ┬**s***_i_* ∈ Q, ┬**p***_j_* ∈ P, with *f*: ℝ^3×6×3^ →ℝ. From this, the general formulation of the proposal can be written as:

(9) $$s\hat{r}p(q_{k})=\sum_{\forall s_{i}\in Q}w(s_{i})\sum_{\forall p_{j}\in P}f(s_{i},p_{j},q_{k})$$

where *sr̂p*(**q***_k_*) is the model estimation of *srp*(**q***_k_*), and the weights *w*(**s***_i_*) will be non-zero if there is an acoustic source in the given position **s***_i_*, or 0 if otherwise.

The basis functions *f*(**s***_i_*, **p***_j_*, **q***_k_*) must be designed so that they provide accurate estimations of the behavior of the real *SRP-PHAT* value at location **q***_k_*, taking into account that there is an active source at position **s***_i_* and that the signal is acquired by the microphone pair **p***_j_*. This generic formulation allows for models (basis functions) as complex as required, in principle able to include any of the considerations described in Section 3.3.

In the experimental work described in Section 5, we are using a relatively simple model that is able to clearly outperform standard *SRP-PHAT* results. In our experiments, the basis functions *f*(**s***_i_*, **p***_j_*, **q***_k_*) describe if point **q***_k_* belongs to the hyperbolic region generated by an acoustic source **s***_j_* and a given pair of microphones **p***_j_*:

(10) $$f(s_{i},p_{j},q_{k})=\{\begin{array}{llll} 1 & if & \left|\Delta \tau (p_{j},s_{i})-\Delta \tau (p_{j},q_{k})\right|\leq \in & \in \geq 0\\ 0 & \text{otherwise} \end{array}$$

where threshold *∈* accounts for the fact that in real-world scenarios there are uncertainties in measuring time delays as discussed in Section 3.3. Using *∈* > 0, the width of the hyperbolic region is not constant, modeling the effect that can be clearly seen in Figure 3(a). In fact, the width increases with distance to the microphone pair, partly because for a given uncertainty (error) in the time delay estimation (due to the fact that we are using sampled signals), the spatial uncertainty (error in precisely assigning a correlation value to a given spacial location) increases as we consider positions further away from the microphone pair generating the hyperbolic region.

The model described by Equations (9) and (10) is valid to reproduce *SRP-PHAT* measurements, as the hyperbolic regions of the power maps are related to the high values of the Generalized Cross Correlation function of each pair of microphones [9]. Consequently the position of the hyperbolic regions is consistent with the time difference of arrival for each microphone pair given a certain speaker position.

### 3.5. Description of a Linear Model of *SRP-PHAT*

Using the model previously proposed in Equation (9) over all positions inside Q the following vector **ŷ** is defined:

(11) $$\begin{array}{cc} \hat{\text{y}}={\left(\begin{array}{ccc} s\hat{r}p(q_{1}) & \cdots & s\hat{r}p(q_{Q}) \end{array}\right)}^{⊤} & q_{k}\in Q \end{array}$$

This section shows that vector ŷ can be represented as a linear combination of vectors of size *Q*. Each vector is only representative of a specific spatial location where an acoustic source can be active. As was described in previous sections, this model accounts for the fact that single acoustic sources are viewed in *SRP-PHAT* data as the intersection of multiple hyperbolic regions.

For each position **q** ∈ Q, define the following vector **v**(**s**):

(12) $$\begin{array}{cccc} v(s)={\left(\upsilon (s,q_{1}),\cdots ,\upsilon (s,q_{Q})\right)}^{⊤} & \text{with} & \upsilon (s,q_{i})=\frac{1}{N}\sum_{\forall p_{j}\in P}f(s,p_{j},q_{i}), & q_{i}\in Q \end{array}$$

where *N* is the number of microphone pairs, *Q* is the size of Q and *f*(**s**, **p***_j_*, **q***_i_*) ∈ ℱ are the basis functions defined in Equation (10).

**Figure 4.**
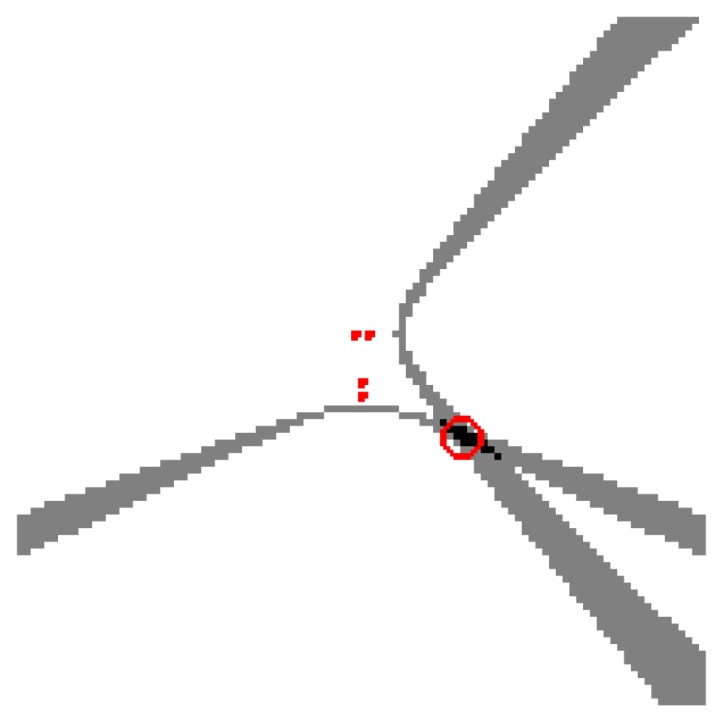
Model content defined for a single active speaker located in the position of the red circle.

Vector **v**(**s**) can be intuitively seen as the ideal *SRP-PHAT* measurements that would be obtained for a single acoustic source located at position s. If Q contains points with constant height, **v**(**s**) can be visualized as an image, composed as the sum of hyperbolic areas (one for each pair of microphones), intersecting at point s (see Figure 4). It must be remarked that v is normalized by definition, *i.e.*, max(**v**(**s**)) = 1.

The proposed generative model consists of the following linear system:

(13) $$\begin{array}{cccc} \hat{y}=Mx & \text{with} & M=\left(\begin{array}{ccc} v(s_{1}) & \cdots & v(s_{Q}) \end{array}\right) & s_{i}\in Q \end{array}$$

where **x** = (*x*_1_, ċ, *x_Q_*)^┬^ is a vector of size *Q*, representing a numerical weight associated to each position considered in set Q, where an acoustic source could be active. In fact, weight *x_i_* corresponds exactly to weight *w*(**s***_i_*) defined in Equation (9) up to a scale factor. In this case, **x** are the unknown parameters of the model.

**Figure 5.**
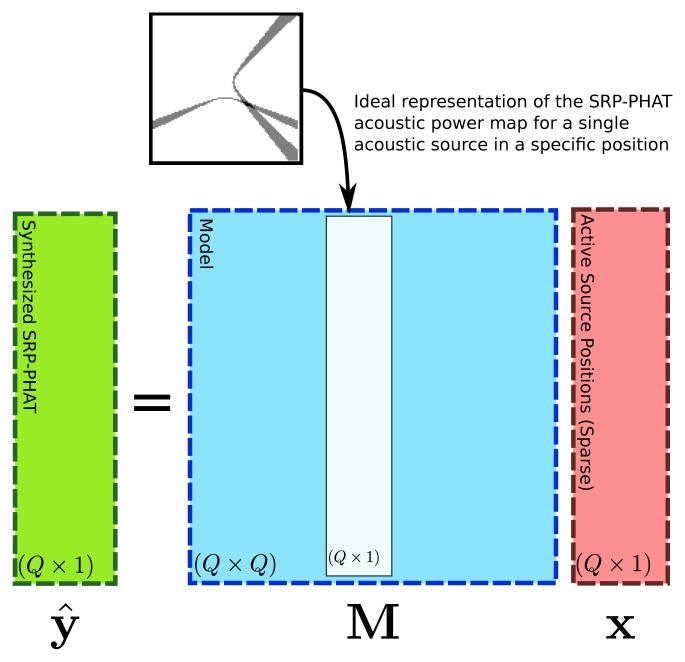
Explicit matrix layout for the model proposal given by Equation (13).

Matrix M is a *Q* × *Q* matrix whose columns are obtained using vector **v** defined at every **s** ∈ Q. Vector **ŷ** can be seen as the *SRP-PHAT* data synthesized by the proposed model as a function of weight vector **x**. Figure 5 shows a graphical diagram of the proposed linear model.

Expanding the terms in Equation (13), vector **ŷ** is obtained as the following weighted sum of vectors:

(14) $$\hat{y}=x_{1}v(s_{1})+\cdots +x_{Q}v(s_{Q})$$

where it is explicitly seen that weight *x_i_* directly affects the influence of vector **v**(**s***_i_*) in the output vector **ŷ**. Therefore, if vector **x** has high values around a single position **s***_i_*, the resulting vector **ŷ** will have a maximum at **s***_i_*, producing a *SRP-PHAT* image consistent with the model presented in the previous section. Nevertheless, as it was discussed in Section 3.3, it must be recalled that the hyperbolic model defined by Equation (10) is only a rough simplification of the real phenomenon, where noise, reverberation and array directivity issues produce artifacts in the *SRP-PHAT* approximation that are not considered in the model. The consideration of these additional effects in the formulation of the basis functions can lead to improvements in the modeling ability of the proposed solution.

## 4. Model Fitting

This section explains how to use the linear model proposed in the previous section to fit real *SRP-PHAT* data. One of the main contribution of the paper is to show that as a result of model fitting, the performance of *SRP-PHAT* based localization techniques can be remarkably improved.

Suppose that vector **y** contains *SRP-PHAT* measurements (arranged in a column vector) obtained in a real scenario:

(15) $$\begin{array}{cc} y={\left(\begin{array}{ccc} \text{srp}(q_{1}) & \cdots & \text{srp}(q_{Q}) \end{array}\right)}^{⊤} & q_{i}\in Q \end{array}$$

with *srp*(**q***_i_*) defined in Equation (1).

Our aim is finding a vector **x** capable of explaining **y** using model **M**. It is expected that **y** includes modeling errors, reverberation, array directivity effects, and noise, thus making the proposed model invalid for an exact representation of **y**. Instead, the goal will be finding a vector **x** capable to *better* explain **y**. The notion of which vector **x** is better at modeling **y** can be answered using optimization techniques.

The basic approach is then to solve the following optimization problem:

(16) $$\underset{x}{\min}\rho (y,\hat{y})=\underset{x}{\min}\rho (y,Mx)$$

where *ρ* is a metric measuring how different are the measurements **y** and the vector **ŷ** generated by the model (*i.e.*, **Mx** from Equation (13)). A straightforward and somehow natural choice for *ρ* is to use the Euclidean distance as a metric:

(17) $$\underset{x}{\min}\;{\left\|y-Mx\right\|}_{2}^{2}$$

which yields to a linear least squares problem. If matrix **M** has full rank, the minimum of Equation (17) is unique and can be obtained in closed-form. Otherwise a regularized problem can be solved instead using Tikhonov regularization [45]. In either case, solving problem (17) represents a weak approach when the model **M** is not accurate enough to fit the data **y**, which contains noise and effects that cannot be reproduced by the model.

The approach of this paper, and one of the basis of our contribution, is to include additional constraints into Equation (17) able to give meaningful answers for **x** with noisy measurements, and for relatively simple basis functions in the generative model. Two basic improvements of problem (17) are proposed and detailed next.

### 4.1. Adding Sparse Constraints

In this paper it is assumed that there is only a small number of simultaneous active acoustic sources inside the space defined by Q, which is a reasonable assumption in the majority of scenarios considered. Given that values of **x** represent positions in which there is an active acoustic source, it is thus sensible to force **x** to have as many zeroes as possible. In the mathematical language that means to force the vector **x** to be a *sparse* vector, in which the number of non-zero elements is limited. In the optimization scheme, making the **x** vector to be *as sparse as possible* is equivalent to forcing the *l_0_* norm of **x** to be minimum.

Finding the vector **x** that simultaneously reduces the error between the input data and the model and forces **x** to be as sparse as possible can be mathematically expressed as follows:

(18) $$\begin{array}{ccc} \underset{x}{\min}{\left\|x\right\|}_{0} & \text{s}.\text{t}. & {\left\|y-Mx\right\|}_{2}^{2}<\eta \end{array}$$

where *η* is a real value that bounds the amount of error and model mismatch that is admissible. Minimizing (18) is very difficult as the *l*_0_ norm makes the problem highly non-linear, NP-Hard and non-convex. No practical method guarantees the global convergence in this case.

Sparse optimization methods have received remarkable attention from the scientific community. Despite its theoretical complexity, several methods and approximations have been proposed so far, and of special relevance are those methods based on using the *l*_1_ norm as a convex relaxation of the *l*_0_ norm [33,46]. This relaxation transforms (18) into the following:

(19) $$\begin{array}{ccc} \underset{x}{\min}\;{\left\|x\right\|}_{1} & \text{s}.\text{t}. & {\left\|y-Mx\right\|}^{2}<\gamma \end{array}$$

where *γ* is an hyperparameter closely related to *η* in (18). Equivalently, problem (19) can be expressed in its Lagrangian form:

(20) $$\underset{x}{\min}\;{\left\|y-Mx\right\|}_{2}^{2}+\lambda {\left\|x\right\|}_{1}$$

where *λ* is the Lagrange multiplier and has a direct relationship with *γ*.

Both Equations (19) and (20) are equivalent convex problems, in which convergence is guaranteed and can be solved in polynomial time.

The problem of finding a least squares estimation subject to a *l*_1_ restriction has been independently presented and popularized under the names of *Least Absolute Shrinkage Selection Operator (LASSO)* [47] and *Basis Pursuit Denoising* [32], being object of intensive study. In the past few years numerous optimization methods have been proposed, some of them adapted to specific problems.

Additionally, several generic libraries and toolboxes implementing those methods have been developed and are being extensively used. The results shown in the paper have been generated using one of these libraries [48], using a truncated Newton interior-point method, described in [49].

Solving the relaxed problem (20) does not necessary imply finding the solution to the original *l*_0_ problem. The closeness and validity of *l*_1_ relaxations have been extensively studied [33]. In some problems, the structure of matrix **M** and the expected degree of sparsity in the solution can make *l*_1_ relaxations to be exact. For general linear systems, as it is the case in this paper, where matrix M has no apparent structure, *l*_1_ relaxation empirically tends to impose only approximate sparse solutions. This paper provides strong experimental evidence of the improvements obtained by imposing *l*_1_ penalties, effectively making the solution **x** more sparse. Sparsity is a strong “prior” that helps to bias the solution **x** so that the effect of noise and model mismatches are properly attenuated.

### 4.2. Adding Subspace Filtering

Although sparsity is a well founded constraint and the *l*_1_ relaxations are effective, the experimental results in Section 5 show that, given the current model, sparsity is not strong enough to cope with errors and model mismatches in real *SRP-PHAT* measurements so that additional strategies must be used to improve model fitting.

This section introduces a new constraint on the problem based on filtering out the part of the input signal **y** that is not reproducible using model **M**.

First decompose **y** into two parts:

(21) $$y=\hat{y}+\tilde{y}=Mx+\tilde{y}$$

where **ŷ** is a term that can be explained exactly by the generative model (*i.e.*, there exists a vector **x** such that **ŷ** = **Mx**) and **ỹ** represents the non-reproducible part of the signal (*i.e.*, **ỹ** ≠ **Mx** for any vector **x**). This section proposes to use subspace filtering to remove the non-reproducible part **ỹ** from the input vector **y**.

First, matrix **M** is expressed using *singular value decomposition* (SVD) as follows:

(22) $$M=U\sum V^{*}$$

where **U** and **V** are unitary matrices of dimensions *Q* × *Q* and **Σ** is a semidefinite positive diagonal matrix of dimension *Q* × *Q*. The diagonal elements of **Σ** are the singular values, sorted in descending order. Using singular values it is possible to know the amount of degrees of freedom available in the model by just looking how many non-zero singular values it has.

By identifying the number of zero singular values of **M**, namely *N_z_*, the SVD decomposition shown in Equation (22) can be expressed using the following sub-matrices:

(23) $$M=\left(\begin{array}{cc} U_{1} & U_{0} \end{array}\right)\left(\begin{array}{cc} \sum_{1} & 0\\ 0 & 0 \end{array}\right)\left(\begin{array}{c} V_{1}^{*}\\ V_{0}^{*} \end{array}\right)=U_{1}\sum_{1}V_{1}^{*}$$

where **U**_0_ and **V**_0_ are *Q* × *N_z_* matrices, **U**_1_ and **V**_1_ are of size *Q* × (*Q* − *N_z_*) and **Σ**_1_ is a diagonal (*Q* − *N_z_*) × (*Q* − *N_z_*) matrix.

**U**_1_ and **U**_0_ are subspace projection matrices. Any nonzero vector **z** such that $U_{1}^{⊤}z=0$ is a vector that cannot be obtained using the model **M**, *i.e.*, **z** ≠ **Mx** for any possible **x**.

So, recalling that U*U = UU* = **I**, both sides of the equality (21) can be multiplied by U* with the following result:

(24) $$\left(\begin{array}{c} U_{1}^{*}y\\ U_{0}^{*}y \end{array}\right)=\left(\begin{array}{c} \sum_{1}^{*}V_{1}^{*}\\ 0 \end{array}\right)x+\left(\begin{array}{c} U_{1}^{*}\tilde{y}=0\\ U_{0}^{*}\tilde{y} \end{array}\right)$$

By definition, if **ỹ** cannot be expressed by the model, then its projection using matrix $U_{1}^{⊤}$ must be zero. Contrary, the projection into the kernel subspace represented by **U**_0_ is nonzero.

Therefore, in order to remove the dependence of **ỹ**, only the Mahalanobis distance of the upper part of system (24) is optimized, regularized with the *l*_1_ term, and resulting into the problem (20) to become:

(25) $$\underset{x}{\min}\;{\left\|\sum_{1}^{-1}U_{1}^{⊤}y-V_{1}^{*}x\right\|}_{2}^{2}+\lambda {\left\|x\right\|}_{1}$$

In practice, a small threshold *ψ* is used to decide if a singular value can be considered zero. Experiments are carried out in Section 5.5 to learn the value of parameter *ψ* from real *SRP-PHAT* data, which turns out to be an important parameter in practice. In order to give meaningful discrete values to *ψ* this paper uses the following ratio:

(26) $$r(\psi )=\frac{\sum_{\lambda_{j}>\psi }\lambda_{j}}{\sum_{i=1}^{Q}\lambda_{i}}100$$

where *diag*(**Σ**) = (*λ*_1_, ċ, *λ_Q_*)^┬^ are the singular values of **M**. The meaning of Equation (26) is basically the percentage of Frobenius norm that **M** has lost after filtering out small singular values using *ψ*. By bounding the ratio with an *energy* threshold, namely *e_ψ_* ∈ [0%, 100%], which can be chosen easily with independence of scale factors (e.g., *e_ψ_* = 50% means half of the energy in the model), the value of *ψ* can be chosen adequately as:

(27) $$\begin{array}{ccc} \underset{\psi }{\min} & \text{s}.\text{t}. & r(\psi )\leq e_{\psi } \end{array}$$

In Section 5, the value of *ψ* is chosen by giving values to *e_ψ_* using (27) afterwards.

After setting to zero all the $N_{z}^{\prime }$ singular values below threshold *ψ*, we can build new matrices **U**′_0_ and ${\text{V}\prime }_{0}(Q\times N_{z}^{\prime })$, **U**′_1_ and ${\text{V}\prime }_{0}(Q\times (Q-N_{z}^{\prime }))$ and ${\sum \prime }_{1}((Q-N_{z}^{\prime })\times (Q-N_{z}^{\prime }))$, for which the SVD decomposition (23) becomes:

(28) $$M\prime =\left(\begin{array}{cc} {U\prime }_{1} & {U\prime }_{0} \end{array}\right)\left(\begin{array}{cc} {\sum \prime }_{1} & 0\\ 0 & 0 \end{array}\right)\left(\begin{array}{c} {V\prime }_{*}^{1}\\ {V\prime }_{*}^{0} \end{array}\right)={U\prime }_{1}{\sum \prime }_{1}{V\prime }_{*}^{1}$$

and the optimization problem (25) becomes:

(29) $$\underset{x}{\min}\;{\left\|{\sum^{\prime }}_{1}^{-1}{U^{\prime }}_{1}^{⊤}{y-V^{\prime }}_{1}^{*}x\right\|}_{2}^{2}+\lambda {\left\|x\right\|}_{1}$$

#### 4.3. Improving *SRP-PHAT* with Model Fitting

The main objective of the paper is to show that, as a result of the optimization methods proposed before, the solution **x** can be used to improve source localization, comparing with traditional approaches directly using *SRP-PHAT* measurements. The detection of local maxima in *SRP-PHAT* acoustic power maps is the standard way to retrieve the position of the acoustic source. This technique yields good results but is still prone to errors due to reverberation and noise and when the number of microphones is limited.

**Figure 6.**
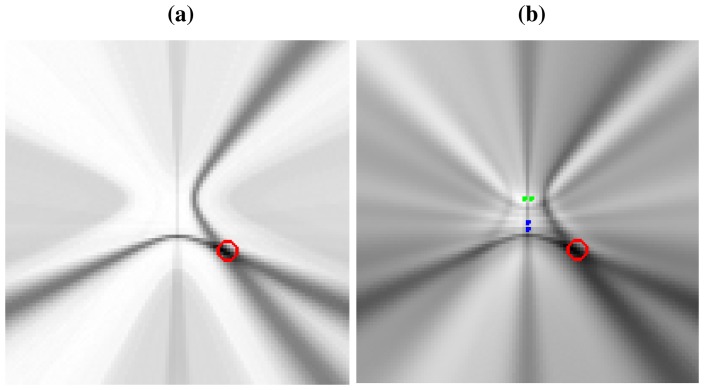
Comparison between real *SRP-PHAT* power map and its denoised version, (a) Denoised acoustic power map described by **ŷ**′; (b) Real *SRP-PHAT* acoustic power map described by **y**.

Our approach consists of replacing the original *SRP-PHAT* measurements **y** with those generated by the model solving the optimization (29), *i.e.*, **ŷ**′ = **M**′**x**′, where **M**′ is obtained from Equation (28) and **x**′ is the solution of Equation (29). Vector **ŷ**′ can also be interpreted as a filtered/denoised version of **y** that is consistent with the proposed model. Figure 6 shows the acoustic power map described by the denoised vector **ŷ**′ (Figure 6(a)) and the original *SRP-PHAT* acoustic power map **y** (Figure 6(b)). From the figure, it seems clear that the *denoising* effectively reduces the number of artifacts and unwanted effects exhibited by the original map, and the assumption is that this *denoised* version **ŷ**′, if properly constrained during the optimization, is a better place to find local maxima truly representing active acoustic sources. In Section 5 the paper gives strong experimental indicators to support this idea.

### 5. Experiments and Discussion

#### 5.1. Experimental Setup

We have evaluated our proposal using the audio recordings of the AV16.3 database [50], an audio-visual corpus recorded in the *Smart Meeting Room* of the IDIAP research institute, in Switzerland.

**Figure 7.**
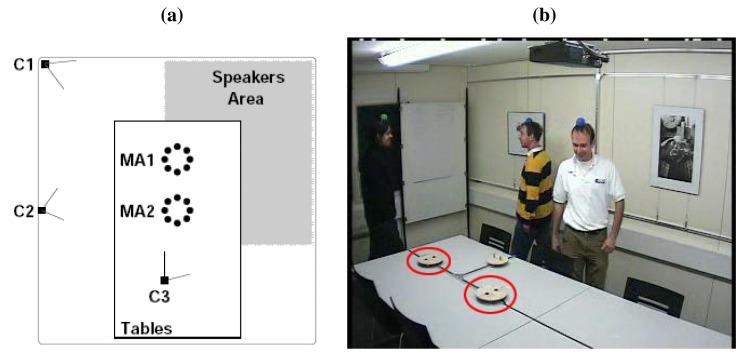
Idiap Smart Meeting Room for AV16.3 recordings (a) Room layout showing the microphone positions in two circular arrays (MA1 and MA2), three cameras (C1, C2 and C3), and the L-shaped area for speaker locations in the recordings, (b) Sample of recorded video frame.

The IDIAP Meeting Room consists on a 8.2 m × 3.6 m × 2.4 m rectangular room containing a centrally located 4.8 m × 1.2 m rectangular table, on top of which two circular microphone arrays of 0.1 m radius are located, each composed by 8 microphones. The centers of the two arrays are separated by 0.8 m and the origin of coordinates is located in the middle point between the two arrays. Possible speakers' locations are distributed along a L-shaped area around the table as seen in Figure 7(a). A detailed description of the meeting room can be found in [51].

The audio recordings are synchronously sampled at 16 KHz, and the complete database along with the corresponding annotation files containing the recordings ground truth is fully accessible on-line at [52]. It is composed by several sequences or recordings which range in the number of speakers involved and their activity. In this paper we will just focus on the single static speakers sequences, whose main characteristics are shown in Table 1. We will refer to the sequences as *seq01*, *seq02* and *seq03* for brevity.

Every audio sequence is assigned a corresponding annotation file containing the real ground truth positions (3D coordinates) of the speaker's mouth at every time frame in which that speaker was talking. The segmentation of acoustic frames with speech activity was first checked manually at certain time instances by a human operator in order to ensure its correctness, and later extended to cover the rest of recording time by means of interpolation techniques. The frame shift resolution was defined to be 40 ms.

#### 5.2. Evaluation Metrics

Our localization algorithm yields a set of spatial coordinates **q**(*t*) = (*x*, *y*, *z*)^┬^ that are estimations of the actual speaker position, for every time frame *t*. These position estimates will be compared, by means of the Euclidean distance, to the ones labeled in a transcription file containing the real positions **s**(*t*) (*ground truth*), of the speaker.

We have decided to use the metrics developed under the CHIL project and described in their Evaluation Plan [53]. A complete description of the CHIL Evaluation strategies can be found at [53], but in this work we will only refer to the *Multiple Object Tracking Precision* (*MOTP*), calculated as the average localization error for all (*N_T_*) acoustically active frames in the data set: $\text{MOT}P=\frac{\sum_{t=1}^{N_{T}}\left\|q(t)-s(t)\right\|}{N_{T}}$.

#### 5.3. Evaluation Plan

We are evaluating our model in a 2D scenario, considering the acoustic power maps generated by *SRP-PHAT* at locations Q belonging to a plane 61 cm above the microphone array positions (this height roughly corresponds to the average height of the speaker positions in the AV16.3 sequences). Locations for *SRP-PHAT* data are calculated uniformly sampling Q in a 10 cm × 10 cm grid.

The procedure to generate the position estimations **q**(*t*) consists of searching for maximum values in vector **ŷ**′ (calculated as described in Section 4.3) that could be seen as a *denoised* version of the original *SRP-PHAT* acoustic power map.

**Figure 8.**
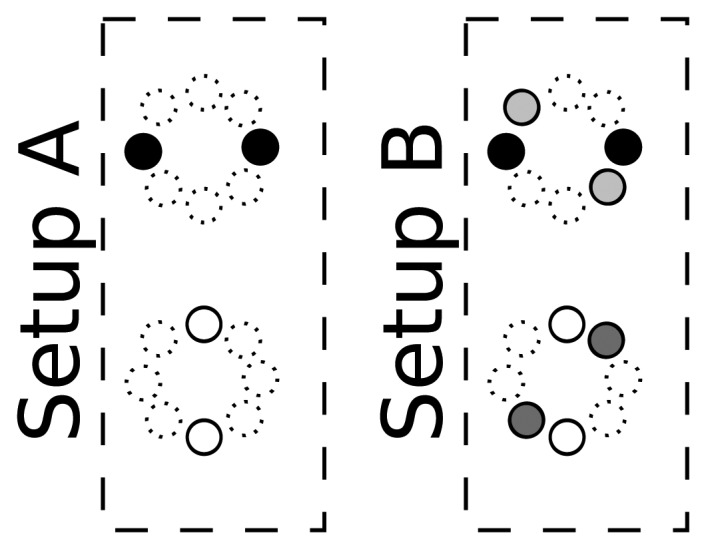
Microphone pairs setups used in the experiments (microphones with the same color belong to the same pair).

In the experimental results shown below, we are assessing the performance of our proposal in terms of:

- Optimization parameters: We will provide results depending on the two main tunable parameters of the optimization algorithms used, namely *λ* and *e_ψ_*.
- The estimation of the optimal values for this parameters will be done on an independent data set (training set) and applied to unseen data in the evaluation stage (test set).
- Sensor array configuration: In this work, we are using a simple microphone array configuration, aimed at evaluating our proposal in a resource-restricted environment. In order to do so, we are using 4 or 8 microphones (out of the 16 available in the AV16.3 data set), grouped in two or four microphone pairs to generate the baseline *SRP-PHAT* acoustic maps. The selected microphone pairs configurations are shown in Figure 8, in which microphones with the same color are considered as belonging to the same microphone pair. Given that the microphone separation for each microphone pair is 20 cm, we will violate spatial aliasing requirements, considering the signal bandwidth. Fortunately, when using *SRP-PHAT*, the use of more than one microphone pair alleviates this problem, as side lobes are different for each pair, and thus their effects are partially compensated.
- Acoustic frame size: We will provide results depending on the length of the acoustic frame, for 80, 160 and 320 ms, to precisely assess to what extent the improvements are consistent with varying acoustic time resolutions.

The baseline we are comparing with will be the results of directly searching the maximum of the *SRP-PHAT* acoustic power map. The position of this maximum will correspond to the most probable source location.

Comparisons will specifically consider the relative improvement in *MOTP*, defined as $\Delta_{r}^{\text{MOT P}}=\frac{\text{MOT}P_{\text{baseline}}-\text{MOT}P_{\text{proposal}}}{\text{MOT}P_{\text{baseline}}}$.

Our main interest is assessing whether the results and improvements are consistent across different conditions. After describing the baseline results (in Section 5.4) and in order to evaluate the generalization capability of the proposed methods, we will address an initial study using sequence *seq01* as the *training set* (in Section 5.5). From this study, we will decide on the optimal values of the tunable parameters used in the optimization process (those leading to the best results), and then use them to provide final performance and improvement results on the *test sets*, namely *seq02* and *seq03* (in Section 5.6). This evaluation plan ensures adequate independence and variability between train and test sets, with different speakers in all sequences (also differing in gender and height).

In all cases were appropriate, we will include references to statistical confidence values for a 95% confidence level, to adequately assess whether the improvements are statistically significant.

#### 5.4. Baseline Results

Tables 2 and 3 show the baseline results using the standard *SRP-PHAT* algorithm for all sequences and different frame sizes, and the two microphone setups of Figure 8.

The main conclusions for the baseline results are:

- The performance obtained is reasonable if we take into account that only two or four microphone pairs are used. Best *MOTP* values are around 50 cm.
- Performance improves as the frame size increases, as expected, given that longer frames lead to better estimations of the correlation functions.
- Adding an additional microphone pair in setup B as compared with setup A also leads to performance improvements as expected.

#### 5.5. Evaluation of the Sensitivity to λ and e_ψ_ Values

The proposed model fitting strategies heavily depend on the estimation of adequate values for both *λ* and *e_ψ_* (as they are the parameters controlling the optimization process), so that a detailed study on the sensitivity of the performance with variations in these parameter values is mandatory

*λ* expresses the relative importance of the sparse constraints applied in the optimization problems (20), (25) and (29), so that the higher its value becomes, the sparser the solution will be. In the *l*_1_ optimization software used [48], it is required that *λ* < *λ_max_* being *λ_max_* dependent on both the model and the input data [49]. In the results shown, the hyperparameter is represented normalized with respect to the calculated *λ_max_*: *λ_norm_* = *λ*/*λ_max_*, as described in [49].

The energy threshold *e_ψ_* used in the subspace filtering strategy described by Equation (27) decides the size of the model that is not able to adequately *explain* the input signal.

To decide on the optimal *λ_norm_* and *e_ψ_* to be used, we will select the values that achieve the best result in terms of *MOTP*, for every microphone setup and frame size.

**Figure 9.**
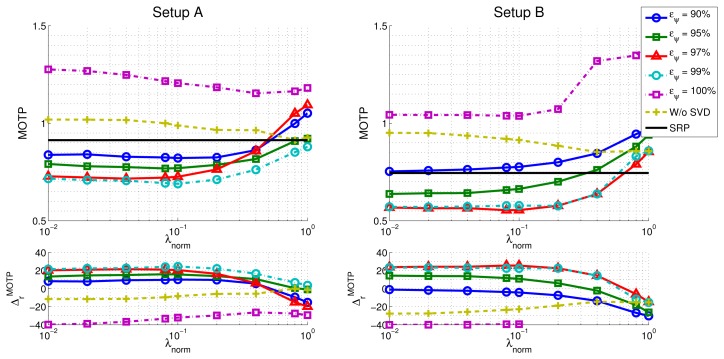
Optimization results for *MOTP* and relative improvements as a function of *λ*_norm_ and *e_ψ_*, for microphone setups A and B on sequence *seq01*. The black trace is the baseline *SRP-PHAT* system.

In the upper part of Figure 9, we show the evolution of the *MOTP* quality metric as a function of *λ_norm_* and the energy value *e_ψ_*, for both microphone setups, evaluating the training sequence *seq01*, with a frame size of 160 ms, as an example. The horizontal black trace show the baseline results for the *SRP-PHAT* algorithm (obviously independent of *λ_norm_* and *e_ψ_*,). In the lower part of Figure 9 the evolution of the relative improvements in *MOTP* are shown.

Additionally, and in order to evaluate the effectiveness of the subspace filtering step, we ran an experiment in which only the optimization with sparse constraints described in Equation (20) is applied (*i.e.*, our proposal without using subspace filtering). The results are shown in the “W/o SVD” trace of Figure 9.

**Figure 10.**
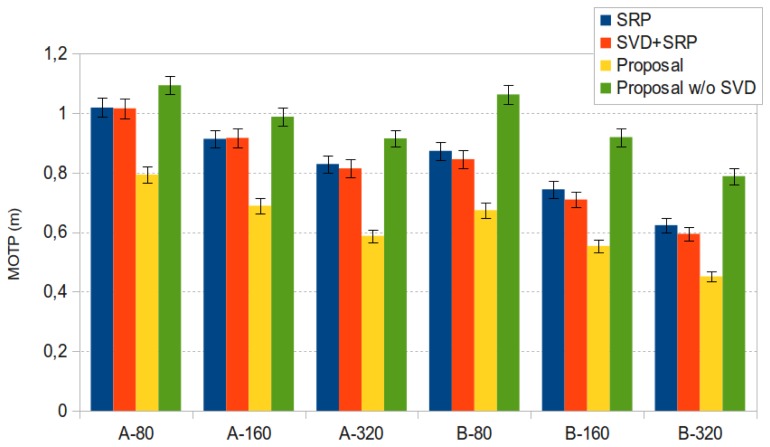
Best *MOTP* results for sequence *seq01* for both microphone setups (A, B) and all frame sizes (80, 160 and 320 ms.), with 95% confidence intervals.

In Figure 10 we show the best *MOTP* results for sequence *seq01* for both microphone setups and all frame sizes, with 95% confidence intervals. Data includes results for the baseline *SRP-PHAT* results (“SRP” in the legend, blue bars), for our proposal (“Proposal” in the legend, yellow bars), and for our proposal without applying the SVD step (“Proposal w/o SVD” in the legend, green bars) (the orange bar (“SVD+SRP” in the legend) refers to results that will be discussed later).

From this, we can conclude that, for adequate values of the optimization tuning parameters:

- Our proposal is able to improve the *SRP-PHAT* results with statistically significant relative improvements of up to almost 25%, with consistent improvements for a wide range of *λ_norm_* values.
- Microphone setups have a similar impact in the relative performance improvements. The improvements for setups A and B are 24.6% and 25.6%, respectively.
- In what respect to the dependency of the best results with *λ_norm_* (once selected the optimal *e_ψ_*), both microphone setups show a desirable behavior, achieving a reasonably clear optimal area for a wide range of parameter values.
- Using either the model with sparse constraints (*i.e.*, “Proposal w/o SVD”) or SVD without actually filtering (*i.e.*, *e_ψ_* = 100%) is giving worse localization results than the *SRP-PHAT* baseline algorithm. It thus seems that fitting the complete model to data is not making any progress even if sparse constraints are included. The explanation of this phenomenon was partially advanced in Section 4.2 but it needs some additional justification. The model that is proposed in this paper is not able to explain every *SRP-PHAT* map (*i.e.*, matrix **M** is rank-deficient). When using any of the optimization strategies proposed in the paper, the position of speakers is the result of looking at local maxima in the *SRP-PHAT* map reproduced through the model. Therefore, in theory, the results must not be necessarily equal to the baseline algorithm, even if subspace filtering is removed, or the *l*_1_ term is not having strong influence. Empirical data tell us that in these cases, localization results can be in fact worse than the baseline. The main result of the paper is to show through experiments that statistically significant improvements can be reached using a specific combination of subspace filtering and sparse constraints. In these cases the model is able to adequately filter the effects of noise and reverberation in the *SRP-PHAT* map, giving a cleaner image about the real position of the speaker.

Table 4 shows the highest relative improvements obtained for sequence *seq01* and the optimal values of the parameters found to achieve these best results $\left(\text{namely}\lambda_{\text{norm}}^{\text{optimal}}\;\text{and}\;e_{\psi }^{\text{optimal}}\right)$. The table shows how the maximum improvement is high and consistent along different frame sizes and microphone setups. Improvements in *MOTP* clearly increase as the frame size increases.

Interestingly, the optimal values for the parameters controlling the optimization process are identical for all frame sizes in the setup A $\left(\lambda_{\text{norm}}^{\text{optimal}-A}=0.1\;\text{and}\;e_{\psi }^{\text{optimal}-A}=99\%\right)$. This seems not to be the case for setup B, in which $e_{\psi }^{\text{optimal}-B}=97\%$ in all cases, but $\lambda_{\text{norm}}^{\text{optimal}-B}$ values varies for different frame sizes. However, even in this case, the improvements are stable for a wide range of parameter values as can be seen in the first three rows of Table 5, where the relative improvements have been calculated for different values of *λ_norm_* (0.04, 0.08 and 0.1), setting $e_{\psi }=e_{\psi }^{\text{optimal}-B}=97\%$.

From Table 4 it also seems that the optimal values of the parameters are dependent on the microphone setup used, as both $\lambda_{\text{norm}}^{\text{optimal}}$ and $e_{\psi }^{\text{optimal}}$ are different for setups A and B. A more detailed evaluation shows that, again, the improvements are stable even when we use the optimal values estimated for setup A $\left(\lambda_{\text{norm}}^{\text{optimal}-A}=0.1\text{and}e_{\psi }^{\text{optimal}-A}=99\%\right)$, in the optimization process for setup B data, as it can be seen in the last row of Table 5.

**Figure 11.**
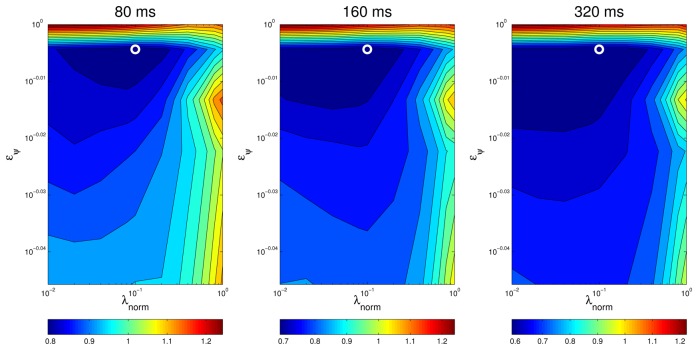
Optimization map for microphone setup A on sequence *seq01*. The circle is the position of the best parameter combination.

**Figure 12.**
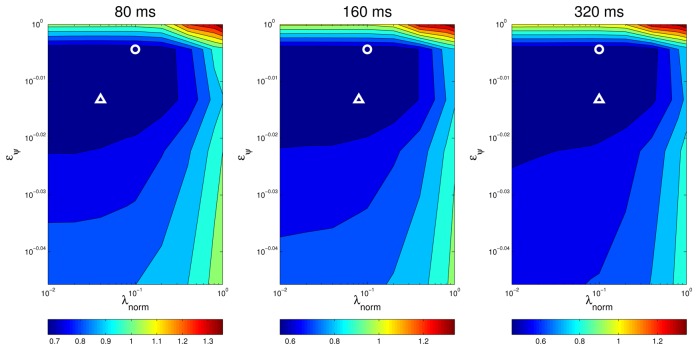
Optimization map for microphone setup B on sequence *seq01*. The circle is the position of the best parameter combination in *seq01* calculated for setup A and the triangle is the position of the best parameter combination in *seq01* calculated for setup B.

An additional way of visually assessing to what extent the results of the optimal values for the optimization parameters are consistent for different situations is plotting a surface map of *MOTP* versus variations on *λ_norm_* and *e_ψ_* and making a comparison. For example, Figures 11 and 12 show this *optimization map* for microphone setups A and B respectively, using sequence *seq01*. In these maps, the optimal points for each evaluation are represented with a *circle* for *seq01* and setup A, and with a *triangle* for *seq01* and setup A. The maps show a *similar* structure for the optimal region in both microphone setups, supporting the idea that the optimal optimization parameters do not heavily depend on changes of the experimental conditions. Moreover, in the cases for microphone setup B, where the optimal points (triangles) seem not to be close to the optimal points of setup A (circles), it can be seen that these positions *belong* to an area with roughly the same *MOTP* level (the area can be recognized as a *flat* optimal region).

The main conclusion of these experiments is that, for the given experimental setup, our proposal is able to clearly outperform the standard *SRP-PHAT* results. The statistically significant relative improvements roughly vary between 22% and 30%, and, what is more important, with little sensitivity to the optimization parameters selected when changing the microphone setup and the frame size used (once the optimal parameters have been estimated for the training data).

To further evaluate the contribution of the subspace filtering strategy, we ran an experiment in which we applied the subspace filtering to the original *SRP-PHAT* data, that is, projecting the *SRP-PHAT* acoustic power map on the span of model **M**′ obtained from (28). This projection generates a new filtered power map, calculated as $y^{*}=U\prime {U\prime }_{⊤}^{1}y$. The results applying this transformation are given in the orange bars of Figures 10 and 15, referred to as “SVD+SRP”. In these figures, we can see that SRP+SVD also outperforms SRP, although the differences are not statistically significant.

#### 5.6. Evaluation on the *Test Set*

The evaluation carried out in the previous section only addresses the estimation of the optimal parameters for a single *training* sequence and the proposal evaluation on this same data set (*seq01*). We still need to assess whether the optimal values estimated for the *training* data set are able to achieve good results when using different sequences. As stated above, we are using *seq02* and *seq03* as independent *test sets*.

**Figure 13.**
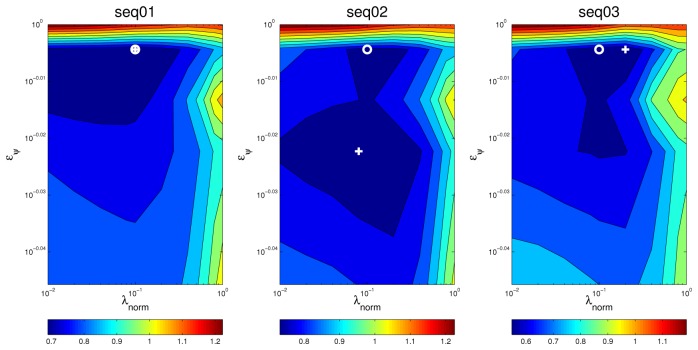
Optimization map for microphone setup A on all sequences, evaluating *MOTP* and frame size 160 ms. The circle is the position of the best parameter combination calculated for sequence *seq01* and the cross is the best position calculated for each sequence.

**Figure 14.**
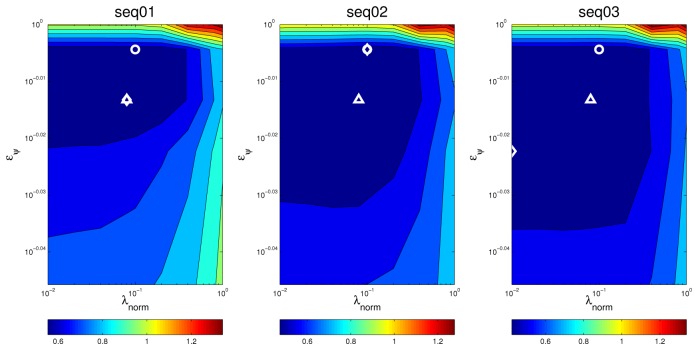
Optimization map for microphone setup B on all sequences, evaluating *MOTP* and frame size 160 ms. The circle is the position of the best parameter combination calculated for sequence *seq01* with setup A,the triangle is the position of the best parameter combination in *seq01* with setup B and the diamond is the best position calculated for each sequence.

Figures 13 and 14 show the *optimization maps* for all sequences, frame size 160 ms, and microphone setups A and B, respectively. The *cross* is located in the optimal point for each sequence and setup A, and the *diamond* is located in the optimal point for each sequence and setup B. It can be seen that, again, the *structure* of the optimal regions are reasonably similar, thus suggesting that the optimal values for the optimization parameters estimated in the training set will also achieve good results in the test sets. The position of the optimal points in each map also belong to the same *flat* optimal region.

**Figure 15.**
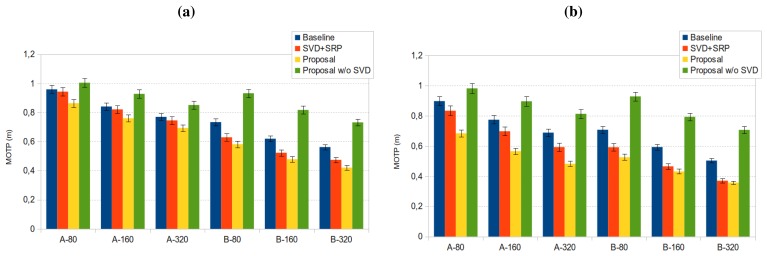
Best *MOTP* results for both microphone setups (A, B) and all frame sizes (80, 160 and 320 ms), with 95% confidence intervals, (a) for sequence *seq02* and (b) for sequence *seq03*.

Figure 15 shows the best *MOTP* results for sequences *seq02* and *seq03* for both microphone setups and all frame sizes, with 95% confidence intervals (using the optimal parameter values estimated for the training sequence *seq01*).

Tables 6 and 7 show the relative improvements achieved when evaluating sequences *seq02* and *seq03* for both microphone setups, also using the optimal parameter values for sequence *seq01*. As expected, the relative improvements are in the range of those obtained for sequence *seq01*, except for sequence *seq02* and microphone setup A (with lower improvements of around 10%). Our hypothesis is that the fact that this is a female speaker imposes significant differences in the speech signals, thus modifying the correlation functions used in the input data, and posing additional difficulties to the optimization process when only two microphone pairs are used. Nevertheless, this will have to be evaluated in future work.

Apart from the case of *seq02* with setup A, the improvements are relevant and statistically significant, roughly varying between 20% and 30%. These achievements also show little sensitivity to the optimization parameters selected, in spite of the fact that we are additionally dealing with different speakers.

### 6. Conclusions and Future Work

This paper has proposed a novel method to localize active acoustic sources in a room equipped with sensor arrays. Two main contributions can be highlighted: First, a simple but very promising generative linear model is proposed to explain *SRP-PHAT* data taken from any geometrical combination of microphone arrays. The model simply reflects the geometry of three-dimensional points sharing common difference of time-of-arrival between each microphone pair. This model is independent of the spectrum properties of the signals emitted by the source and can be easily computed in practice. Second, this paper shows, using convincing experiments based on publicly available data, that such a simple model can be used to fit real *SRP-PHAT* data that is usually very noisy and has many unmodeled effects (such as reverberation in the scene). Fitting the model is done by imposing two constraints. The first one is forcing the model parameters to be sparse, as acoustic sources cannot be densely distributed in a typical environment. The second constraint simply removes the part of the measurements that is not exactly reproducible by the model. In the light of the experimental results, these two constraints in combination are the real key of the paper, notably improving the performance of state-of-the-art localization methods based on *SRP-PHAT*. It is also worth mentioning that all algorithms and experiments proposed in the paper are very easy to reproduce.

In future works the performance of this approach must be thoroughly validated in rooms with multiple speakers and using the whole three-dimensional set of spatial positions. Immediate improvements should cover all issues commented in Section 3.3. That means to propose basis functions in the model that take into account additional factors, such as the spectral content of the acoustic sources, directivity pattern effects in the microphone arrays, and also adding geometric information that would help to predict reverberation effects. The authors believe that improvements in the model may yield remarkable improvements in the localization accuracy in real world scenarios.

## Figures and Tables

**Table 1.** Characteristics of the audio sequences used in the experimental results.

Sequence name	speaker	Average speaker height* (m)	duration(s)	number of ground truth frames
seq01-1p-0000	male	54.3	208	2,248
seq02-1p-0000	female	62.5	171	2,411
seq03-1p-0000	male	70.3	220	2,636

* In the reference coordinate system.

**Table 2.** Baseline *MOTP*(*m*) results for all sequences, different frame sizes and microphone setup A.

		80 ms	160 ms	320 ms
seq01	*MOTP*	1.02 ± 0.03	0.91 ± 0.03	0.83 ± 0.03
seq02	*MOTP*	0.96 ± 0.03	0.84 ± 0.03	0.77 ± 0.02
seq03	*MOTP*	0.90 ± 0.03	0.77 ± 0.03	0.69 ± 0.03

**Table 3.** Baseline *MOTP*(*m*) results for all sequences, different frame sizes and microphone setup B.

		80 ms	160 ms	320 ms
seq01	*MOTP*	0.87 ± 0.03	0.74 ± 0.03	0.62 ± 0.02
seq02	*MOTP*	0.73 ± 0.02	0.62 ± 0.02	0.56 ± 0.02
seq03	*MOTP*	0.71 ± 0.02	0.59 ± 0.02	0.50 ± 0.01

**Table 4.** Relative improvements of *MOTP*(*m*) for sequence *seq01*, including the values of the optimal parameters, estimated per microphone setup and per frame size.

		80 ms	160 ms	320 ms

setup A	$\Delta_{r}^{\text{MOT P}}$	22.1%	24.6%	29.1%
	$\lambda_{\text{norm}}^{\text{optimal}}$	0.1	0.1	0.1
	$e_{\psi }^{\text{optimal}}$	99%	99%	99%

setup B	$\Delta_{r}^{\text{MOT P}}$	22.9%	25.6%	27.6%
	$\lambda_{\text{norm}}^{\text{optimal}}$	0.04	0.08	0.1
	$e_{\psi }^{\text{optimal}}$	97%	97%	97%

**Table 5.** Relative improvements of *MOTP*(*m*) for sequence *seq01* and microphone setup B, using different values for the optimization parameters.

		80ms	160ms	320ms

setup B	$\Delta_{r}^{\text{MOT P}}$	22.9%	24.2%	26.7%
$e_{\psi }^{\text{optimal}-B}=97\%$	*λ_norm_*	0.04	0.04	0.04

setup B	$\Delta_{r}^{\text{MOT P}}$	22.1%	25.6%	27.2%
$e_{\psi }^{\text{optimal}-B}=97\%$	*λ_norm_*	0.08	0.08	0.08

setup B	$\Delta_{r}^{\text{MOT P}}$	22.2%	25.3%	27.6%
$e_{\psi }^{\text{optimal}-B}=97\%$	*λ_norm_*	0.1	0.1	0.1

setup B	$\Delta_{r}^{\text{MOT P}}$	21.4%	22.6%	24.3%
$\lambda_{\text{norm}}^{\text{optimal}-A}=0.1e_{\psi }^{\text{optimal}-A}=99\%$				

**Table 6.** Relative improvements of *MOTP*(*m*) for sequence *seq02*, using the optimal parameter values estimated for sequence *seq01*.

		80 ms	160 ms	320 ms

setup A	$\Delta_{r}^{\text{MOT P}}$	10.0%	9.6%	9.9%
	*λ_norm_*	0.1	0.1	0.1
	*e_ψ_*	99%	99%	99%

setup B	$\Delta_{r}^{\text{MOT P}}$	20.7%	22.9%	25.1%
	*λ_norm_*	0.04	0.08	0.1
	*e_ψ_*	97%	97%	97%

**Table 7.** Relative improvements of *MOTP*(*m*) for sequence *seq03*, using the optimal parameter values estimated for sequence *seq01*.

		80 ms	160 ms	320 ms

setup A	$\Delta_{r}^{\text{MOT P}}$	23.8%	26.9%	29.9%
	*λ_norm_*	0.1	0.1	0.1
	*e_ψ_*	99%	99%	99%

setup B	$\Delta_{r}^{\text{MOT P}}$	25.7%	27.3%	29.0%
	*λ_norm_*	0.04	0.08	0.1
	*e_ψ_*	97%	97%	97%
